# Glucose-Assisted One-Pot Hydrothermal Synthesis of Hierarchical-Structured MoS_2_/C Quasi-Hollow Microspheres for High-Performance Lithium Ion Battery

**DOI:** 10.3390/polym13050837

**Published:** 2021-03-09

**Authors:** Xingang Liu, Jiang Tan, Xi Li, Chuhong Zhang

**Affiliations:** State Key Laboratory of Polymer Materials Engineering, Polymer Research Institute, Sichuan University, Chengdu 610065, China; liuxingang@scu.edu.cn (X.L.); jiangtan202103@163.com (J.T.); nicylixi@163.com (X.L.)

**Keywords:** MoS_2_, hydrothermal synthesis, hollow microsphere, lithium ion battery (LIB)

## Abstract

In this work, hierarchical MoS_2_/C quasi-hollow microspheres are prepared by a one-pot hydrothermal process with the addition of glucose. The glucose is not only inclined to form the roundish sphere in the completion of the synthesis of MoS_2_, but at the same time the microspheres formed by the glucose can act as the nuclei on which the MoS_2_ grows. Glucose, acting as a nucleating agent, has the advantages of being low-cost and environmentally friendly, which can simplify the fabrication process. The interiors of the MoS_2_/C samples are multi-hole and quasi-hollow, which is beneficial for the insertion and extraction of lithium ions. For the first time, we demonstrate that hierarchical-structured MoS_2_/C quasi-hollow microspheres exhibit an excellent cycling stability and rate capability in lithium ion batteries (LIBs) and are significantly superior to the bulk MoS_2_. The method presented in this article may provide a simple, clean. and economical strategy for the preparation of MoS_2_/C microspheres as a feasible and promising anode material for LIBs.

## 1. Introduction

New energy technology is considered to be one of the five major technical areas in the development of the 21st century world economy, which receives great attention around the world. Rechargeable lithium ion batteries (LIBs) are currently key components of portable, entertainment, computing, and telecommunication equipment due to their light weight, high specific capacity, low self-discharge rate, good cycling performance, having no memory effect, and being eco-friendly [1,2]. Due to the limited lithium storage capacity of the commercial graphite anode (372 mAh g^−1^) in LIBs, they can no longer suit the fast growth of the modern society. Therefore, the need to explore alternative anode materials with a smaller size, lighter weight, and higher reversible capacity is more demanding than ever [3,4].

As a typical type of layered transition metal dichalcogenide (TMD), MoS_2_ has a sandwich structure, stacked by S–Mo–S tri-layers through Van Der Waals interaction [5,6]. This unique structure endows MoS_2_ with numerous applications in the fields of lubricants [7], catalytic hydrodesulfuration [8], hydrogen storage [9], evolution reactions [10,11], and anode materials for Li^+^/Na^+^/Mg^2+^ batteries [12,13,14,15,16,17,18]. Following the first patent of MoS_2_ as the anode material in LIBs, a large number of MoS_2_ with different morphologies (such as nanoflake, nanoflower, hierarchical microsphere) were fabricated for LIBs [5,19,20,21]. As an anode material, MoS_2_ has a specific capacity of up to 1000 mAh g^-1^ [22], but its cycling stability is unsatisfactory due to the great volume expansion-induced mechanical strain upon the cycling, which subsequently causes electrode pulverization and the loss of active material/current collector integrity [6,23,24]. On the other hand, the relatively low electric conductivity of bulk MoS_2_ also poses a negative effect on its rate capability in LIBs.

To address the above issues, two approaches have been proposed [25]. One is to devise special nanostructures of MoS_2_ (such as nanotubes [26], nanoflakes [27], and mesoporous [28] and hierarchical micro-nanostructures [29,30]) so that they can release the mechanical strain of the volume change and lessen the barrier for the Li^+^ transport. However, the aggregation of nano-structured MoS_2_ may still happen during the charge/discharge process and it will still suffer from a low electric conductivity. The other approach is to fabricate the MoS_2_ composites with some electrically conductive carbons or polymers, which obviously is beneficial in terms of improving the electrical conductivity of the electrode material [31,32,33,34,35,36,37,38]. Therefore, the combination of these two strategies—i.e., the preparation of a MoS_2_/electrically conductive carbon composite, in which MoS_2_ presents a special morphology—may offer a perfect solution to the problems of bulk MoS_2_, as mentioned above. Recently, hierarchical hollow nanoparticles have aroused great interest because they not only effectively increase the electrolyte/electrode contact area and shorten the pathway of ion diffusion, but also buffer the volume expansion of electrodes, thus stabilizing the electrode structure under the charge–discharge process. Although MoS_2_/C hollow microspheres have been reported once [25,29], the involvement of mono dispersed polystyrene microspheres as templates makes the synthetic process expensive and sophisticated. 

Glucose, a widely accessible biochemical, is a source of carbon, which is also considered a method that adds value to biorefinery. In this paper, we present a fast, simple, and low-cost method to prepare MoS_2_/C quasi-hollow microspheres using a one-pot hydrothermal method with the assistance of glucose. Compared with other hydrothermal methods to prepare molybdenum disulfide/carbon composites [34,35], we selected ammonium molybdate and thiourea as the raw materials to synthesize MoS_2_ at the molecular level, which can make the glucose molecules first aggregate into nuclei and then cause ammonium molybdate and thiourea to grow in situ on the surface. First, the MoS_2_ layer grows through heterogeneous nucleation on the surface of the spherical core formed by glucose aggregation. Second, the glucose is pyrolyzed into porous carbon in the interior to form the MoS_2_/C microspheres with a quasi-hollow structure after heat treatment. As expected, the MoS_2_/C quasi-hollow microspheres can not only improve the cycling stability of MoS_2_, but also enhance the electric conductivity of the electrode material, resulting in an excellent rate performance in LIBs.

## 2. Experimental Section

### 2.1. Synthesis of Hierarchical MoS_2_/C Quasi-Hollow Microspheres

Ammonium molybdate tetrahydrate ((NH_4_)_6_Mo^VI^_7_O_24_·4H_2_O, 99%) and thiourea (C(NH_2_)_2_S, 99%) were purchased from Shanghai Aladdin Co., Ltd. Glucose was purchased from Kelong Chemical Co., Ltd. (Chengdu, China). All the reagents were analytical grade and directly used as received without further purification. 

In a typical procedure, 8.6 mmol/L of ammonium molybdate, 140 mmol/L of thiourea, and 148 mmol/l of glucose were dissolved in 30 mL of distilled water. After being stirred for 30 min, the solution was then transferred into a Teflon-lined stainless steel autoclave. The autoclave was sealed and maintained at 240 °C for 48 h. The black precipitates were collected by filtering, washing with distilled water, and freeze-drying for 24 h. Subsequently, the as-prepared black powder was fabricated into MoS_2_/C composites by annealing in an Ar atmosphere under 800 °C for 2 h. The content of glucose varied between 148 and 296 mmol/L (MoS_2_/C composites obtained with the addition of 148, 222, and 296 mmol/L of glucose) and such obtained composites are denoted as MoS_2_/C-1, MoS_2_/C-2, and MoS_2_/C-3, respectively, according to the approximate mass ratio of glucose and the total weight of the ammonium molybdate and thiourea. The bulk MoS_2_ was prepared through the same procedure without the addition of glucose for comparison. 

### 2.2. Characterizations 

Powder X-ray diffraction (XRD) patterns were recorded on a Rigaku (Smart lab III) of Hitachi Company (Tokyo, Japan) using Cu Kα radiation within 2θ = 10°–80° with a scanning rate of 10°/min. Product morphologies were observed by field emission scanning electron microscopy (FESEM) and high-resolution transmission electron microscopy (TEM) (Philips-FEI company Hillsboro, OR, USA). X-ray photoelectron spectra (XPS) were collected on an Escalab 250Xi X-ray photoelectron spectrometer (Thermo Fisher Scientific Co., Ltd., Waltham, Ma, USA). 

### 2.3. Electrochemical Measurements 

The anodes were fabricated by mixing active materials (MoS_2_, MoS_2_/C composites) with acetylene black carbon and polyvinylidene fluoride (PVDF), at a weight ratio of 8:1:1 in the solvent of *N*-methyl-2-pyrrolidone (NMP). The obtained slurry was cast onto a Cu foil with a wet film thickness of about 150 μm and dried at 120 °C in a vacuum for 12 h. The electrodes were punched to φ=13 mm discs before use. Subsequently, CR 2032 coin-type cells were assembled in an argon-filled glove box using MoS_2_, or MoS_2_/C composites as the working electrode, Li foil as the counter electrode, a glass fiber membrane (Whatman 1825-025) as a separator, and 1M of LiPF_6_ dissolved in a 1:1 mixture of ethylene carbonate and diethyl carbonate as the electrolyte. The cells were measured using an automatic battery tester system of Wuhan Landian Electronics Co., Ltd. (Land CT2001A, Wuhan, China) and galvanostatic charged and discharged at various current densities in the voltage range of 0.01–3.0 V. The cyclic voltammetry (CV) was carried out in the potential range of 0.01–3.0 V (vs. Li/Li^+^) with a scan rate of 0.2 mV/s, and the electrochemical impedance spectroscopy (EIS) was measured by applying an AC voltage of 10 mV in the frequency range of 100 kHz to 0.01 Hz using the Biologic VMP3 electrochemical workstation.

## 3. Results and Discussion

### 3.1. Characterization of Structure and Morphology 

**Figure 1:**
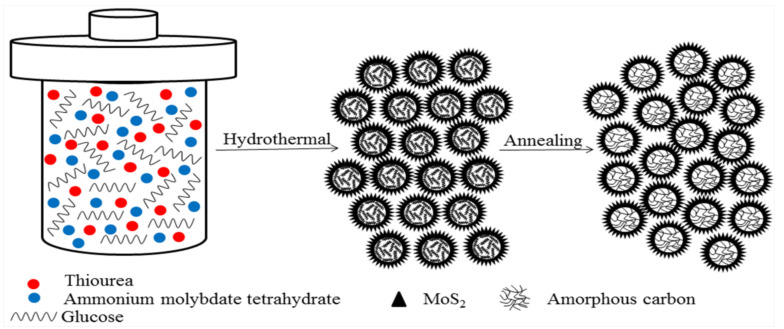
Schematic illustration of the formation mechanism of the hierarchical MoS_2_/C quasi-hollow microspheres.

Figure 1 is the schematic illustration of the formation of hierarchical MoS_2_/C quasi-hollow microspheres. First, glucose spontaneously aggregates, acting as a spherical nuclei onto which MoS_2,_ synthesized by the reaction of (NH_4_)_6_Mo^VI^_7_O_24_·4H_2_O (Mo source) and C(NH_2_)_2_S (S source), grows through heterogeneous nucleation. In the process of hydrothermal reaction, glucose molecules undergo dehydration and cross-linking reaction, carbonize to form carbon-carbon bonds, and gradually form carbon microspheres. Subsequently, after the high-temperature annealing treatment, its aromaticity is further increased, which is beneficial to the improvement of conductivity [38,39]. Then, MoS_2_/C quasi-hollow microspheres with hierarchical structures are obtained. 

**Figure 2:**
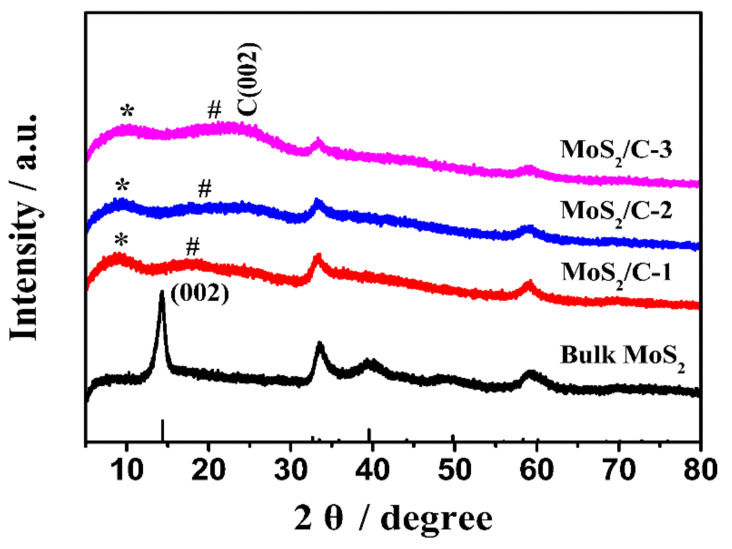
X-ray diffraction (XRD) patterns of the synthesized pristine MoS_2_ and MoS_2_/C composites prepared with different amounts of glucose (a.u. is arbitrary unit).

**Figure 3:**
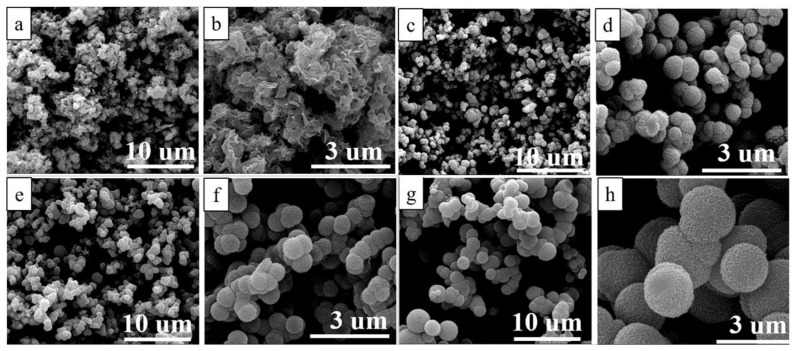
Scanning electron microscopy (SEM) image of (**a**,**b**) pristine MoS_2_, (**c**,**d**) MoS_2_/C-1, (**e**,**f**) MoS_2_/C-2, and (**g**,**h**) MoS_2_/C-3 composites.

Figure 2 shows the XRD pattern of pristine MoS_2_ and MoS_2_/C composites prepared with different amounts of glucose. The XRD pattern of the annealed bulk MoS_2_ can be indexed as the hexagonal crystalized structure of MoS_2_ (JCPDS no. 37-1492), and the strong (002) peak centered at 2θ = 14.2° indicates a well-stacked layered structure. However, in the MoS_2_/C composites with a different carbon content, the (002) plane peak disappears, indicating that the stacking layered structure of MoS_2_ is inhibited in the MoS_2_/C composites 38. The other two peaks at 2θ = 33.0° and 58.9° are in good agreement with those of pristine MoS_2_. The XRD results clearly reveal that the structure of MoS_2_ is mono or has few layers, which is further confirmed by the results of the high-resolution TEM (Figure 3). Besides these, another three weak peaks which do not belong to the MoS_2_ can also be found in the MoS_2_/C composites. The weak peak at 2θ = 25° should be attributed to the (002) plane of amorphous carbon, as the annealing temperature of 800 °C is much lower than the graphitization temperature of 3000 °C. In addition, another two weak peaks, marked with * and #, do not belong to the MoS_2_ or the carbon. According to the Bragg equation, the d-spacing of peak * is 0.97∼1.18 nm and that of peak # is 0.49∼0.58 nm, which do not agree with the d-spacing of MoS_2_ (0.62 nm) or carbon (0.34 nm). Referring to the MoS_2_/amorphous carbon composites synthesized by a hydrothermal route [17,38,40,41], it is known that the d-spacing of peak * is very close to the distance of adjacent MoS_2_ nanosheets in amorphous carbon, suggesting that some amorphous carbon is inserted into the layer of MoS_2_. The d-spacing of # (0.49∼0.58 nm) can be indexed to the distance between the MoS_2_ layer and the carbon layer. Besides these peaks, no other peaks are formed in the MoS_2_/C composites, indicating that impurity does not exist in the MoS_2_/C composites. 

The morphology of the as-prepared pristine MoS_2_ and MoS_2_/C composites were first observed by Scanning electron microscopy (SEM). As shown in Figure 3a, MoS_2_ tend to form a flower-like agglomeration assembled by the MoS_2_ nanosheets. With the introduction of carbon, the MoS_2_/C composite shows a hierarchical structure, as shown in Figure 3c–h, which is composed of MoS_2_ growing on the surface of the C microspheres. This hierarchical structure enlarges the contact area of the anode of the MoS_2_/C and the electrolyte. Additionally, it can shorten the Li^+^ diffusion channel in the charge–discharge process. Compared with the pristine MoS_2_, there was no agglomeration of the MoS_2_ nanosheets in the MoS_2_/C hierarchical structure, suggesting that the amorphous carbon plays a vital role in stabilizing the MoS_2_ nanosheets. At the same time, it can be observed that the MoS_2_/C quasi-hollow microspheres become bigger and more roundish rather than flocking together by increasing the content of amorphous carbon by varying the amount of glucose in the hydrothermal process. By increasing the amount of glucose, it is easier for glucose to aggregate to individually bigger spherical nuclei through the interaction of oxygen-containing functional groups, as do the resulting MoS_2_/C microspheres formed by means of heterogeneous nucleation on glucose.

**Figure 4:**
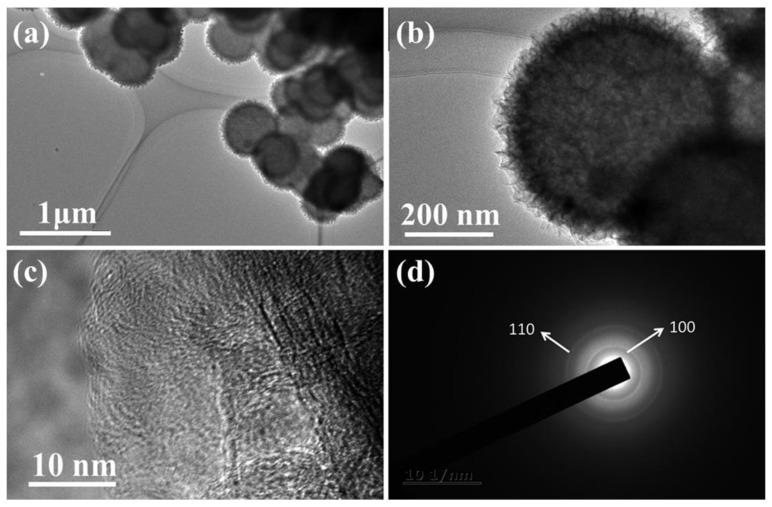
Transmission electron microscope (TEM) images of (**a**–**c**) MoS_2_/C-2 microspheres at different magnifications; (**d**) electron diffraction pattern of MoS_2_/C-2.

To further investigate the hierarchical structures of the MoS_2_/C microspheres, TEM characterization was performed on one of the composites of MoS_2_/C-2. As shown in Figure 4a–d, it is obvious that MoS_2_/C microspheres exhibit a quasi-hollow structure, the center of which is different from its margin: the interior is mainly composed of porous residual amorphous carbon due to the incomplete decomposition of glucose under Ar, while the bulk shell indicates the existence of the MoS_2_. The high-resolution TEM images of the MoS_2_/C microspheres (Figure 4c) reveals that the shells are indeed composed of 1–3 layers of MoS_2_ and the interlayered spacing is about 0.65 nm which is very close to the value calculated from the XRD patterns by using the Bragg equation. A selected area electron diffraction (SAED) pattern of the quasi-hollow MoS_2_/C composite displays two bright diffraction rings belonging to the (100) and (110) reflections of the hexagonal-phase MoS_2_, which is consistent with the XRD results.

**Figure 5:**
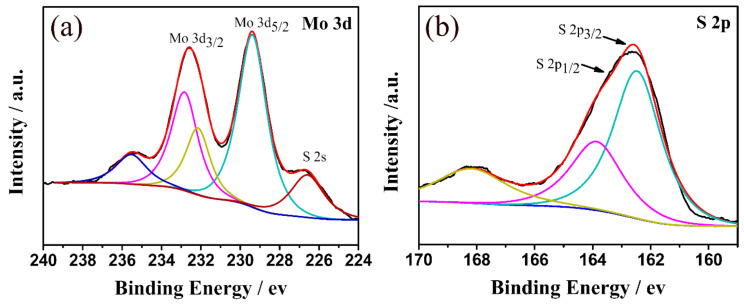
(**a**) Mo 3d spectra and (**b**) S 2p X-ray photoelectron spectroscopy (XPS) spectra of the MoS_2_/C-2 composite.

X-ray photoelectron spectroscopy (XPS) was conducted to determine the chemical composition of the MoS_2_/C composites. The atomic ratio of the Mo and S elements calculated from the XPS spectra is 2.02, approaching the stoichiometric value of MoS_2_ [42]. The Mo 3d XPS spectrum of the MoS_2_/C-2 sample shows two broad peaks at 229.3 eV and 232.4 eV, which can be assigned to the doublet Mo 3d5/2 and Mo 3d3/2, respectively (Figure 5a) [42,43,44]. The S 2p spectrum can be deconvoluted into two peaks centered at 162.3 eV and 163.5 eV, which correspond to S 2p3/2 and S 2p1/2, respectively (Figure 5b) [42,43,44]. Furthermore, the Mo 3d XPS spectrum and S 2p XPS spectrum suggest that Mo^4+^ and S^2-^ are the dominant states in the MoS_2_/C samples [34]. 

### 3.2. Electrochemical Performance Characterization

**Figure 6:**
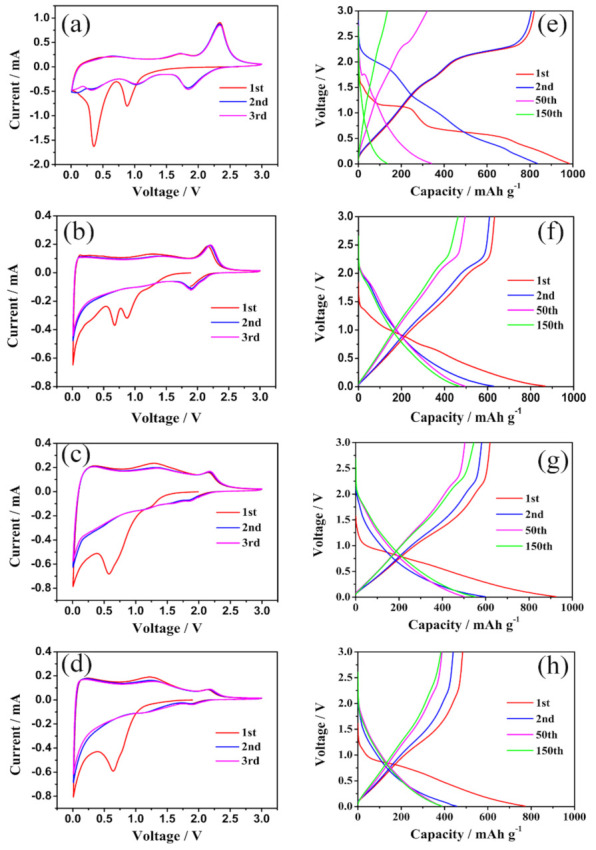
Cyclic voltammograms (CV) of (**a**) pristine MoS_2_, (**b**) MoS_2_/C-1, (c) MoS_2_/C-2, and (**d**) MoS_2_/C-3 composite electrodes; galvanostatic charge/discharge profiles of (**e**) pristine MoS_2_, (**f**) MoS_2_/C-1, (**g**) MoS_2_/C-2, and (**h**) MoS_2_/C-3 electrodes.

Pristine MoS_2_ and MoS_2_/C composites were assembled as a half battery in order to compare their electrochemical properties. Figure 6a–d show the cyclic voltammograms (CV) of pristine MoS_2_ and MoS_2_/C composites at a scan rate of 0.2 mV/s. The CV curves of pristine MoS_2_ show two reductive peaks at 0.8 and 0.3 V and two corresponding oxidation peaks at 1.7 V and 2.2 V in the first cycle. The two reductive peaks at 0.8 V and 0.3 V are due to the reactions (1) and (2) as follows, respectively:

(1) $${\mathrm{MoS}}_{2}+{\mathrm{xLi}}^{+}+{\mathrm{xe}}^{-}\to {\mathrm{Li}}_{x}{\mathrm{MoS}}_{2}$$

(2) $${\mathrm{Li}}_{x}{\mathrm{MoS}}_{2}+\left(4-x\right){\mathrm{Li}}^{+}+\left(4-x\right)e^{-}\to \mathrm{Mo}+2{\mathrm{Li}}_{2}S$$

The pronounced oxidation peaks at 1.7 V and 2.2 V could be attributed to the delithiation of Li_2_S [43,44]. In the second and third cathodic sweeps, a new reductive peak at ∼1.8 V appears, which could be attributed to the following reaction (3):

(3) $$2{\mathrm{Li}}^{+}+S+2e^{-}\to {\mathrm{Li}}_{2}S$$

In contrast, MoS_2_/C composites present a similar but slightly different CV behavior compared to the pristine MoS_2_. During the first anodic scan, there are two peaks located at 0.8 V and 0.6 V in the MoS_2_/C-1 composite, which could be attributed to the reactions (1) and (2) [41,45]. Compared with MoS_2_/C-1, the reductive peak of the MoS_2_/C-2 and MoS_2_/C-3 at 0.8 V becomes inconspicuous for the existence of carbon interlayers between the neighboring MoS_2_ layers preventing the phase transformation of MoS_2_ from trigonal prismatic to octahedral [41]. During the subsequent cathodic scan of the MoS_2_/C-1 electrode, two oxidative peaks at 1.2 V and 2.2 V are observed, which can be attributed to the oxidation of Li_2_S into S [6,25,46]. After the first cycle, the electrode is mainly composed of Mo and S instead of the initial MoS_2_ [6]. In the subsequent scans, the two peaks observed in the first anodic scan disappears. Instead, a new prominent reductive peak at 2.0 V presents, which could be also attributed to the reaction (3) [6,25]. In contrast, in the second scan the anodic peaks at 1.4 V and 2.2 V overlap with those in the first scan, suggesting excellent electrochemical reversibility and stability. The 2.0/2.2 V redox pair constitutes a reversible redox couple, resembling those of sulfur electrodes closely [25].

The galvanostatic discharge/charge profiles of pristine MoS_2_ and MoS_2_/C composites are presented in Figure 6e–h, respectively. As shown in Figure 6e, pristine MoS_2_ presents two potential plateaus at ∼0.3 V and 0.8 V in the first discharge process due to the Li^+^ intercalation reaction followed by a series of conversion reactions. In contrast, there are two inconspicuous potential plateaus at 0.8 V and 0.6 V on the discharge curves of the MoS_2_/C-1 composites, which is caused by the conversion reaction of the Li^+^ intercalation of the MoS_2_. Nevertheless, only one potential plateau at ∼0.6 V presents at the first discharge curve and the plateau corresponding to the Li^+^ intercalation disappears. During the subsequent charge process of the pristine MoS_2_ and MoS_2_/C composites, a series of potential plateaus are all attributed to the oxidation of Li_2_S into S. The information reflected by these charge/discharge curves are in agreement with the results of the CV curves. The initial discharge capacities of pristine MoS_2_, MoS_2_/C-1, MoS_2_/C-2, and MoS_2_/C-3 are 983.6, 867, 924.6, and 771.3 mAh/g, respectively. After 150 continuous charge–discharge cycles, the reversible discharge capacities of pristine MoS_2_, MoS_2_/C-1, MoS_2_/C-2, and MoS_2_/C-3 retain about 134.8, 467.8, 549.7, and 387.9 mAh/g, respectively, suggesting that the electrochemistry performance of the MoS_2_/C composites is superior to that of pristine MoS_2_ and the structural stability of MoS_2_/C-2 is superior among the MoS_2_/C composites. 

**Figure 7:**
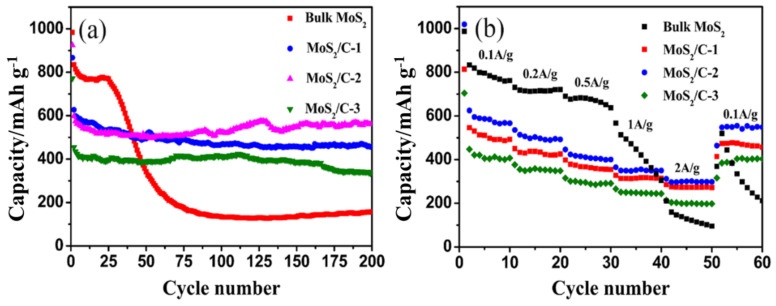
(**a**) Cycling performance of the bulk MoS_2_ and MoS_2_/C electrodes measured in the voltage range of 0.01–3.0 V at a current density of 100 mA g^−1^; (**b**) rate capability of the bulk MoS_2_ and MoS_2_/C electrodes between 0.01 and 3 V at different current densities.

The cycling performances of MoS_2_/C quasi-hollow microspheres are compared in Figure 7. As shown in Figure 7a, the pristine MoS_2_ exhibits an initial discharge capacity of 979.2 mAh g^-1^, which decays quickly within five cycles to 792.2 mAh g^−1^, then after a short plateau of up to 25 cycles continuously drops, and it only can maintain about 100 mAh g^-1^ beyond 75 cycles. However, in contrast, the MoS_2_/C composite electrodes exhibit a significantly improved cycling stability. The initial discharge capacity of the MoS_2_/C electrodes are 867-, 924.6- and 771.3 mAh g^-1^ respectively and after a slight cycle loss in the first five cycles, these electrodes remain with reversible capacities of 456.9, 560.5, and 338.8 mAh g^-1^, respectively, even after 200 cycles. One has to note that the calculated capacity of the composites in which about 62.55%~72.7% of the carbon has no capacity contribution. To make a further comparison, we summarized the electrochemical properties of some of the MoS_2_/C electrodes as shown in Table 1. It can be seen that the reversible specific capacity obtained from the MoS_2_/C-2 is comparable to those of the other kinds of MoS_2_/C electrodes.

Moreover, the MoS_2_/C-2 electrode demonstrates a better rate performance in all the electrodes as shown in Figure 7b. The capacity of MoS_2_/C-2 delivered 586, 506, 410, 350, and 300 mAh g^-1^ at 0.1, 0.2, 0.5, 1, and 2 A g^-1^, respectively. When the current density recovers to 100 mA g^-1^ after cycling under the high current densities, MoS_2_/C-2 sample can still regain a reversible capacity of 550 mA g^-1^. However, the capacity of the bulk MoS_2_ fades continually under the current density of 1 A g^-1^ and drops to ~ 100 mAh g^-1^ at 2 A g^−1^. When the current density recovers to 100 mA g^-1^, the capacity of the bulk MoS_2_ still drops continually, to far below its initial capacity. The superior electrochemical performance of the MoS_2_/C electrode can be attributed to the following reasons. Firstly, the MoS_2_/C quasi-hollow microspheres with a larger specific area can provide a contact area between the electrode material and the electrolyte, which may facilitate the Li^+^ insertion/distraction in the charge/discharge process. Secondly, the interstices between the ultrathin MoS_2_ nanosheets and quasi-hollow interior of the MoS_2_ microspheres play the role of buffering the mechanical stresses induced by the volumetric expansion/shrinkage of the MoS_2_ [25]. Thirdly, the porous carbon cores may serve as adsorbents to restrain the discharge products of the sulfur (lithium polysulfide) from dissolving into electrolytes [25,47]. Lastly, the excessive carbon is not beneficial for the capacity of the MoS_2_/C microspheres but favorable to the cycling stability because the carbon in the MoS_2_/C microspheres is amorphous and it will lower the capacity of the MoS_2_/C microspheres. 

**Figure 8:**
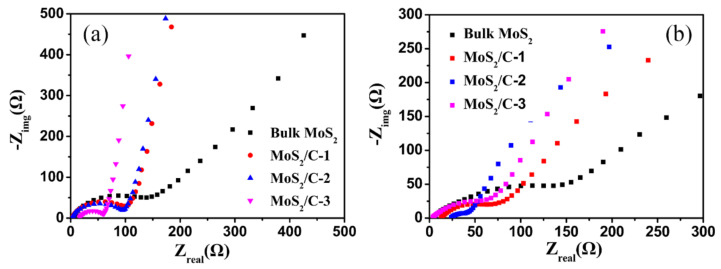
Electrochemical impedance spectra of the bulk MoS_2_ and MoS_2_/C electrodes before cycling (**a**) and after cycling 50 cycles (**b**) at a current density of 100 mA/g.

To gain an in-depth understanding of the good electrochemical performance of the MoS_2_/C sample, the electrochemical impedance spectra (EIS) of the bulk MoS_2_ and the MoS_2_/C electrodes before cycling and after cycling 50 cycles are measured and presented in Figure 8, respectively. Two overlapped depressed semi-circles at high frequency along with an inclined spike at low frequency are observed for all the spectra. The two semi-circles could be attributed to the charge–transfer resistance (R_ct_) between the active material and the electrolyte, and correspond to the lithium diffusion process within the electrodes [38,48,49]. The charge–transfer resistance of the bulk MoS_2_, MoS_2_/C-1, MoS_2_/C-2, and MoS_2_/C-3 is approximately 110, 98, 95, and 60 Ω before cycling, and the corresponding values are about 120, 80, 37, and 50 Ω after the 50th cycle of the charge–discharge process, respectively. It can be seen that the MoS_2_/C-2 composite has a much lower charge–transfer resistance among all the electrodes, which is beneficial for the transmission of Li^+^ and electrons during the charge–discharge process. Furthermore, the much lower impedance of the MoS_2_/C composites demonstrates that the existence of porous carbon can greatly enhance the conductivity of the MoS_2_/C electrode.

## 4. Conclusions

In summary, hierarchical quasi-hollow MoS_2_/C microspheres were successfully prepared by a facile one-pot approach with the assistance of glucose. The as-prepared MoS_2_/C microspheres are significant due to their hierarchical structures integrated with the MoS_2_ nanostructure, in which the aggregation of MoS_2_ is effectively prevented for the porous carbon microspheres. These unique hierarchical quasi-hollow MoS_2_/C microspheres exhibited a greatly improved Li-ion storage performance. In particular, the MoS_2_/C composites exhibited outstanding cycling stability and better rate performance than the bulk MoS_2_. Among all the electrodes, the MoS_2_/C-2 electrode exhibited a higher capacity and better rate performance than the other electrodes. The amorphous carbon in the interior of the MoS_2_/C microspheres can prevent the aggregation of MoS_2_ in the discharge/charge process and promote the conductivity of the MoS_2_/C microspheres simultaneously. However, the excessive amorphous carbon will result in a decrease in the capacity. It can be believed that the facile and low-cost synthesis of quasi-hollow MoS_2_/C microspheres with a better performance has promising applications in the energy storage and conversion system.

## Figures and Tables

**Table 1:** Summary of electrochemistry properties of MoS_2_/C flexible electrodes.

Materials	Current Density mA g^−1^	Cyclic Number	Specific Capacity mAh g^−1^	References
C@MoS_2_ microsphere	100	100	652	[25]
Bowl-like C@ MoS_2_	100	100	798	[31]
Flower-like MoS_2_/C	100	50	834	[35]
MoS_2_-MWCNT	100	30	938	[36]
MoS_2_/C nanoflowers	100	50	888.1	[43]
MoS_2_/C-2	100	200	598	This work
